# Facility-Level Medical Oncology Specialist Availability and First-Line Time to Treatment Failure in Lung Cancer: A Nationwide C-CAT Registry Analysis

**DOI:** 10.3390/curroncol33080462

**Published:** 2026-08-01

**Authors:** Shinya Kajiura, Hironaga Satake, Ryuji Hayashi, Takayuki Yoshino

**Affiliations:** 1 JSMO Secretariat Division, Japanese Society of Medical Oncology, Tokyo 105-0013, Japan; satakeh@kochi-u.ac.jp; 2 Department of Medical Oncology and Palliative Medicine, Toyama University Hospital, Toyama 930-0194, Japan; hsayaka@med.u-toyama.ac.jp; 3 Department of Medical Oncology, Kochi Medical School, Kochi University, Kochi 783-8505, Japan; 4 Board of Directors, Japanese Society of Medical Oncology, Tokyo 105-0013, Japan; 5 President, Japanese Society of Medical Oncology, Tokyo 105-0013, Japan; tyoshino@east.ncc.go.jp; 6 Department of Global Oncology, National Cancer Center Hospital East, Kashiwa 277-8577, Japan

**Keywords:** lung cancer, C-CAT, comprehensive genomic profiling, real-world data, medical oncology, time to treatment failure, treatment-process duration, cancer-care infrastructure

## Abstract

Japan’s Center for Cancer Genomics and Advanced Therapeutics (C-CAT) provides a national platform for studying genomic-medicine-era care. We conducted a nationwide registry/database study to examine whether facility-level Japanese Society of Medical Oncology (JSMO) specialist availability was associated with first-line time to treatment failure (TTF) in lung cancer. The primary exposure was treating-facility JSMO specialist count (0–1 vs. ≥2); dual JSMO/Japanese Respiratory Society availability was a secondary lung-cancer-oriented exposure. First-line TTF was defined from systemic therapy start to recorded first-line treatment end or death, whichever occurred first. The cohort included 5456 patients at 241 facilities: 1477 in the 0–1 group and 3979 in the ≥2 group. Kaplan–Meier estimated median first-line TTF was 4.2 versus 5.1 months (log-rank *p* < 0.001). In the full clinical adjustment model, the HR for ≥2 versus 0–1 specialists was 0.911 (facility-cluster robust 95% CI, 0.806–1.029; robust *p* = 0.133). The point estimate was in the direction of longer recorded treatment-process duration, but the robust confidence interval included no association. Linking C-CAT with facility-level workforce data demonstrates a health-services use of national genomic registry data and supports the development of chemotherapy-specific databases with patient-level, time-anchored clinical information.

## Simple Summary

This study linked Japan’s Center for Cancer Genomics and Advanced Therapeutics (C-CAT) registry with facility-level specialist records to examine first-line treatment duration in lung cancer. At facilities with two or more registry-listed Japanese Society of Medical Oncology specialists, the estimate pointed toward longer time to treatment failure, but the range of estimates after accounting for clustering within facilities included no association. A secondary analysis used availability of physicians listed by both the Japanese Society of Medical Oncology and the Japanese Respiratory Society as a lung-cancer-oriented indicator. Here, time to treatment failure reflects duration of a recorded treatment process; it does not show why treatment ended or whether a longer duration was clinically preferable. Specialist availability may serve as a facility-level indicator of oncology-care infrastructure. By demonstrating how national genomic-registry and workforce data can be linked, this study identifies the patient-level, treatment, and follow-up information needed in future national chemotherapy databases.

## 1. Introduction

Contemporary lung cancer care illustrates how CGP-era oncology requires both molecular testing and coordinated institutional infrastructure. Lung cancer remains one of the largest global cancer burdens and the leading cause of cancer death, with nearly 2.5 million new cases and 1.8 million deaths estimated worldwide in 2022 [1]. Treatment for non-small cell lung cancer has shifted from broadly histology-based approaches toward molecularly stratified systemic therapy integrating targeted agents, immune checkpoint inhibitors, cytotoxic chemotherapy, and increasingly detailed biomarker assessment [2]. Comprehensive genomic profiling (CGP) has become an important component of this landscape by identifying actionable alterations, resistance mechanisms, and potential trial options, especially when conventional companion diagnostics are negative or incomplete [3]. As therapeutic choices, testing strategies, specimen requirements, and sequencing decisions expand, advanced lung cancer care increasingly depends on coordinated infrastructure across respiratory medicine, medical oncology, pathology, molecular diagnostics, radiology, and cancer genomic medicine [2,4,5].

In Japan, CGP has been implemented through a national cancer genomic medicine system under public health insurance, supported by a network of designated cancer genomic medicine hospitals and expert panels [6]. The Center for Cancer Genomics and Advanced Therapeutics (C-CAT) functions as the national datacenter for this system, aggregating clinical and genomic information generated through CGP testing and returning annotated C-CAT Findings reports to participating institutions [7]. This infrastructure has enabled nationwide real-world analyses of cancer genomic medicine, including studies focused on the clinical utility of CGP in non-small cell lung cancer [3]. At the same time, implementation of CGP involves substantial institutional workflows, including administrative coordination, expert-panel preparation, and patient-facing communication [8,9]. Although C-CAT was not designed as a chemotherapy-specific national clinical database, it provides a nationwide opportunity to connect CGP-era lung cancer records with facility-level care-infrastructure indicators [3,7].

Medical oncology specialist availability is one measurable component of that infrastructure. In Japan, the presence and responsibilities of medical oncology departments have expanded in designated cancer care hospitals, particularly in relation to molecularly targeted therapy and immune checkpoint inhibitors [10]. International literature on multidisciplinary lung cancer care suggests that coordinated team-based approaches are associated with differences in staging completeness, treatment timing, treatment receipt, and clinical outcomes in non-small cell lung cancer, although the evidence base is heterogeneous and largely observational [4,5,11]. These observations support evaluating registry-listed Japanese Society of Medical Oncology (JSMO) specialist availability as a facility-level structural signal in contemporary lung cancer care.

We therefore conducted a nationwide C-CAT lung cancer analysis to evaluate whether facility-level registry-listed JSMO specialist availability was associated with first-line time to treatment failure (TTF), an observable treatment-process endpoint in C-CAT [12,13]. Because JSMO specialist count alone may partly reflect hospital scale or broad systemic therapy infrastructure, the Japanese Respiratory Society (JRS) specialist registry was used only to refine the lung-cancer specificity of this facility-level proxy. We evaluated high-confidence dual JSMO/JRS availability as a secondary specificity-oriented proxy for facilities in which JSMO-certified medical oncology expertise may be closer to lung cancer care workflows, such as respiratory oncology conferences and systemic therapy decision-making. Together, these analyses were intended to assess how far current C-CAT data can move from facility-level specialist availability toward the question of medical oncology specialist contribution and to identify chemotherapy-specific data elements needed for future national clinical databases.

## 2. Materials and Methods

### 2.1. Study Design and Data Source

We conducted a nationwide retrospective registry/database analysis using data from the Center for Cancer Genomics and Advanced Therapeutics (C-CAT), the national datacenter for cancer genomic medicine in Japan. C-CAT aggregates clinical and genomic information generated through comprehensive genomic profiling within the Japanese cancer genomic medicine system and enables nationwide real-world analyses of CGP-era oncology practice.

Patient-level C-CAT records were linked to facility-level registry-listed specialist availability variables according to treating facility. The analysis evaluated associations between facility-level specialist availability and first-line time to treatment failure (TTF), and summarized endpoint ascertainment within the registry data.

### 2.2. Study Cohort and Analytic Datasets

Eligible cases were C-CAT-defined lung cancer cases identified using prespecified C-CAT cancer-type and lung histology/subtype variables before endpoint-specific exclusions. Patients were required to have facility information linkable to facility-level specialist availability variables. For the primary analysis, patients were additionally required to have the first-line systemic therapy start date used for endpoint construction and either a recorded first-line treatment end date, death date, or a non-death censoring date. Patients with negative intervals from the first-line start date to the event or censoring date were excluded from the primary endpoint denominator.

The primary analysis cohort was defined by analyzability for first-line TTF. Cohort counts, facility counts, event counts, censoring counts, and exclusions are reported in the Results and Tables. Supportive or supplementary endpoint datasets, if used, were endpoint-specific and were not necessarily identical to the first-line TTF analyzable denominator.

### 2.3. Facility-Level Specialist Availability Variables

The primary exposure was facility-level registry-listed Japanese Society of Medical Oncology (JSMO) specialist count, categorized as 0–1 versus ≥2 registry-listed JSMO specialists. This exposure was assigned at the treating-facility level.

The key secondary exposure was high-confidence dual JSMO/Japanese Respiratory Society (JRS) specialist availability, defined as the presence at the treating facility of at least one registry-listed physician who could be matched as both a JSMO specialist and a JRS specialist. Because JSMO specialist count alone may partly reflect hospital scale or general systemic therapy infrastructure, this secondary variable was evaluated to approximate facility-level availability of JSMO expertise oriented toward lung cancer care. Genome medicine facility category was grouped as core hospital, hub hospital, or liaison hospital.

### 2.4. Primary Endpoint and Censoring

First-line TTF was defined as the time from first-line systemic therapy start to recorded first-line treatment end or death, whichever occurred first. The start date was the recorded first-line treatment start date when available; otherwise, the earliest recorded systemic therapy administration start date was used, consistent with the prespecified endpoint construction rule. Patients without a recorded first-line treatment end or death were censored at the recorded non-death censoring date, including last available survival-confirmation information when applicable. Supportive OS used the same first-line systemic therapy start-date rule as time zero. The earliest parseable death date across outcome records defined the event; otherwise, follow-up was censored at the latest parseable last-survival-confirmation date. In the ordinary Model 5 cohort, 4891 observations used an explicit first-line start and 194 used the prespecified earliest-systemic-administration fallback. Ordinary Model 5 coefficients were reproduced with Breslow ties before facility-cluster robust covariance was calculated with the treating facility code as the cluster. One censored observation in the sparse specimen-type “other” category caused a nonestimable nuisance coefficient; robust Model 5 inference therefore used the estimable 5084-case subset, in which the exposure coefficients were unchanged at the reported precision. For the CGP-timing sensitivity, first-line start remained the time-scale origin and C-CAT registration defined delayed entry; risk intervals were (entry, exit], and registration on or after the exit date was excluded.

Time was calculated in days and converted to months by dividing by 30.4375. Patients with missing start date, missing event and censoring date, invalid patient identifier, missing primary exposure, or negative time interval were excluded from the analyzable endpoint denominator. Zero-time events were retained for Kaplan–Meier analysis and offset by 1 × 10^−6^ months for primary Cox model fitting only; sensitivity analyses replaced zero time with one day, the smallest positive observed interval, or excluded zero-time observations. A lower hazard of the first-line TTF event corresponds to longer observed first-line TTF.

### 2.5. Endpoint Ascertainment and Data Completeness

Endpoint ascertainment was summarized to describe denominator construction and data completeness for first-line TTF within C-CAT. Metrics included start-date availability, first-line treatment end or death event availability, non-death censoring date availability, endpoint analyzability, event and censoring distribution among analyzable cases, negative intervals, and exclusions from the primary denominator. Current TTF event source was classified as recorded first-line treatment end or death without a recorded end. A termination reason was used only from an explicit first-line record ending on the endpoint date; none was inferred. All explicitly labeled first-line records were aggregated for treatment-class mapping. Driver-result timing relative to first-line start was assessed before considering adjustment, without treating unknown/not tested as negative.

### 2.6. Statistical Analysis

Categorical variables were summarized as counts and percentages. Continuous variables were summarized as medians and interquartile ranges, where applicable. Kaplan–Meier methods and log-rank tests were used only to describe unadjusted time-to-event distributions for first-line TTF.

Cox proportional hazards models estimated hazard ratios (HRs) and 95% confidence intervals (CIs) for first-line TTF. For the primary exposure, five model specifications were used: Model 1 was unadjusted; Model 2 adjusted for genome medicine facility category; Model 3 adjusted for genome medicine facility category and treatment start year; Model 4 adjusted for age, sex, Eastern Cooperative Oncology Group performance status, lung histology/subtype, genome medicine facility category, and treatment start year; and Model 5 adjusted for age, sex, Eastern Cooperative Oncology Group performance status, lung histology/subtype, specimen type, panel type, genome medicine facility category, and treatment start year. Models 4 and 5 were complete-case clinical adjustment models.

Because the primary exposure was assigned at the facility level, primary inference used Cox models with facility-cluster robust standard errors based on treating facility code, using the same model specifications and complete-case rules. Conventional confidence intervals and *p* values from ordinary Cox models were retained as secondary comparisons.

Facility volume was defined as the number of C-CAT strict-lung cohort cases at each facility before primary-endpoint analyzability filtering. A Model 5 sensitivity analysis added log2-transformed facility volume. Missingness for Model 5 covariates was summarized by exposure. Because the prespecified histology unknown/missing group combined true blanks with rare histologies, broad or ambiguous labels, typographic variants, and unmapped nonblank values, multiple imputation was not performed; instead, a sensitivity analysis retained the unknown/missing groups as explicit categories.

Statistical analyses were performed using R version 4.5.2 (R Foundation for Statistical Computing, Vienna, Austria).

### 2.7. Ethics and Informed Consent

This study used anonymized C-CAT data. Under the Japanese cancer genomic medicine system, patients provide informed consent before C-CAT registration. This secondary analysis used anonymized C-CAT analysis data obtained through the C-CAT data-use framework. The authors did not access information that could identify individual participants.

The study was approved by the Institutional Review Board of Toyama University on 1 May 2024 (Approval No. R2024016) and by the Information Utilization Review Board of C-CAT in August 2025 (Approval No. CDU2025-010N). Anonymized C-CAT data were accessed for research purposes on 29 January 2026. The study was conducted in accordance with the Declaration of Helsinki, as revised in 2013.

## 3. Results

### 3.1. Study Cohort and Baseline Characteristics

The primary analysis cohort included 5456 patients treated at 241 facilities and was defined by analyzability for first-line TTF. Of these, 1477 patients were treated at 97 facilities with 0–1 registry-listed JSMO specialists, and 3979 patients were treated at 144 facilities with ≥2 registry-listed JSMO specialists (Table 1). The median age was 66 years (IQR, 57–73); 3427 patients (62.8%) were male, and ECOG performance status was 0 in 2150 patients (39.4%), 1 in 2893 (53.0%), and ≥2 in 334 (6.1%). Adenocarcinoma was the most common histology/subtype (3459 patients [63.4%]), followed by other non-small-cell lung cancer (655 [12.0%]), squamous cell carcinoma (607 [11.1%]), and small-cell lung cancer (377 [6.9%]). Tissue specimens were used in 3912 patients (71.7%), and FoundationOne CDx was the most common panel type (3334 [61.1%]).

Genome medicine facility category differed by facility-level JSMO specialist availability. In the overall cohort, 856 patients (15.7%) were treated at core hospitals, 1460 (26.8%) at hub hospitals, and 3140 (57.6%) at liaison hospitals. In the 0–1 JSMO specialist group, most patients were treated at liaison hospitals (1378 [93.3%]), whereas the ≥2 JSMO specialist group included patients treated at core hospitals (856 [21.5%]), hub hospitals (1361 [34.2%]), and liaison hospitals (1762 [44.3%]) (Table 1). High-confidence dual JSMO/JRS availability was present for 2674 patients (49.0%), and combined JSMO/JRS facility availability was present for 4877 patients (89.4%).

In the pre-endpoint C-CAT strict-lung cohort, the facility-level median case count was 9 (IQR, 4–23.75; range, 1–74) among 98 facilities with 0–1 JSMO specialists and 20 (IQR, 8–37; range, 1–269) among 145 facilities with ≥2 specialists. The corresponding patient-weighted medians were 35 (IQR, 17–50) and 48 (IQR, 27–71). Facility volume and JSMO specialist count were moderately correlated (Spearman ρ = 0.342), with substantial overlap in facility volume between exposure groups (Supplementary Table S5).

### 3.2. Primary Endpoint Ascertainment

First-line TTF endpoint ascertainment was evaluated in the pre-endpoint-exclusion C-CAT-defined lung cancer cohort, which included 1580 cases at facilities with 0–1 JSMO specialists and 4158 cases at facilities with ≥2 JSMO specialists (Table 2, Panel A). The corresponding facility counts before endpoint exclusion were 98 and 145; after endpoint exclusions, the primary analyzable facility counts were 97 and 144, respectively.

First-line TTF was analyzable in 1477 of 1580 cases (93.5%) in the 0–1 group and 3979 of 4158 cases (95.7%) in the ≥2 group, which formed the Kaplan–Meier and Cox model denominator. Among analyzable cases, first-line TTF events occurred in 1370 patients (92.8%) in the 0–1 JSMO specialist group and 3597 patients (90.4%) in the ≥2 JSMO specialist group; 107 patients (7.2%) and 382 patients (9.6%), respectively, were censored at the recorded non-death censoring date. Events included recorded first-line treatment end or death before a recorded treatment end. Event-source composition was 1336 recorded first-line treatment ends (90.5%), 34 deaths without a recorded first-line treatment end (2.3%), and 107 censored observations (7.2%) in the 0–1 group, versus 3344 (84.0%), 253 (6.4%), and 382 (9.6%), respectively, in the ≥2 group (Supplementary Table S8).

Negative intervals were uncommon (3 [0.2%] and 8 [0.2%]) and were excluded from the primary endpoint denominator. Overall, 103 cases (6.5%) in the 0–1 group and 179 cases (4.3%) in the ≥2 group were excluded from the primary denominator because of missing dates or negative intervals. The median observed time to event or censoring among analyzable cases was 4.2 months (IQR, 2.3–11.5) in the 0–1 JSMO specialist group and 5.0 months (IQR, 2.5–13.4) in the ≥2 group.

Among 4680 recorded-end events, the explicit source labels were “Invalidation cancellation” in 2370 (50.6%), “Finished as planned” in 1151 (24.6%), cancelation due to side effects in 605 (12.9%), other reasons in 122 (2.6%), and patient request in 46 (1.0%); 386 (8.2%) were unknown, not recorded, or ambiguous. “Invalidation cancellation” is the source label and was not reinterpreted as radiographic progression (Supplementary Table S8).

First-line treatment class was unknown/unclassifiable in 251 patients (4.6%). Non-mutually exclusive flags identified cytotoxic therapy in 4018 (73.6%), targeted therapy in 781 (14.3%), and immune checkpoint inhibitor-containing therapy in 2351 (43.1%). At least one positive result among eight driver fields was documented in 1066 patients (19.5%); 3538 (64.8%) had no documented positive result but at least one explicit negative result, and 852 (15.6%) had neither a positive nor negative result documented. Because biomarker-result dates were unavailable and C-CAT registration followed first-line start in 5417 patients (99.3%), driver status could not be established as a baseline covariate; no regimen/driver-adjusted Cox model was fitted (Supplementary Table S9).

### 3.3. First-Line TTF by JSMO Specialist Availability

**Figure 1:**
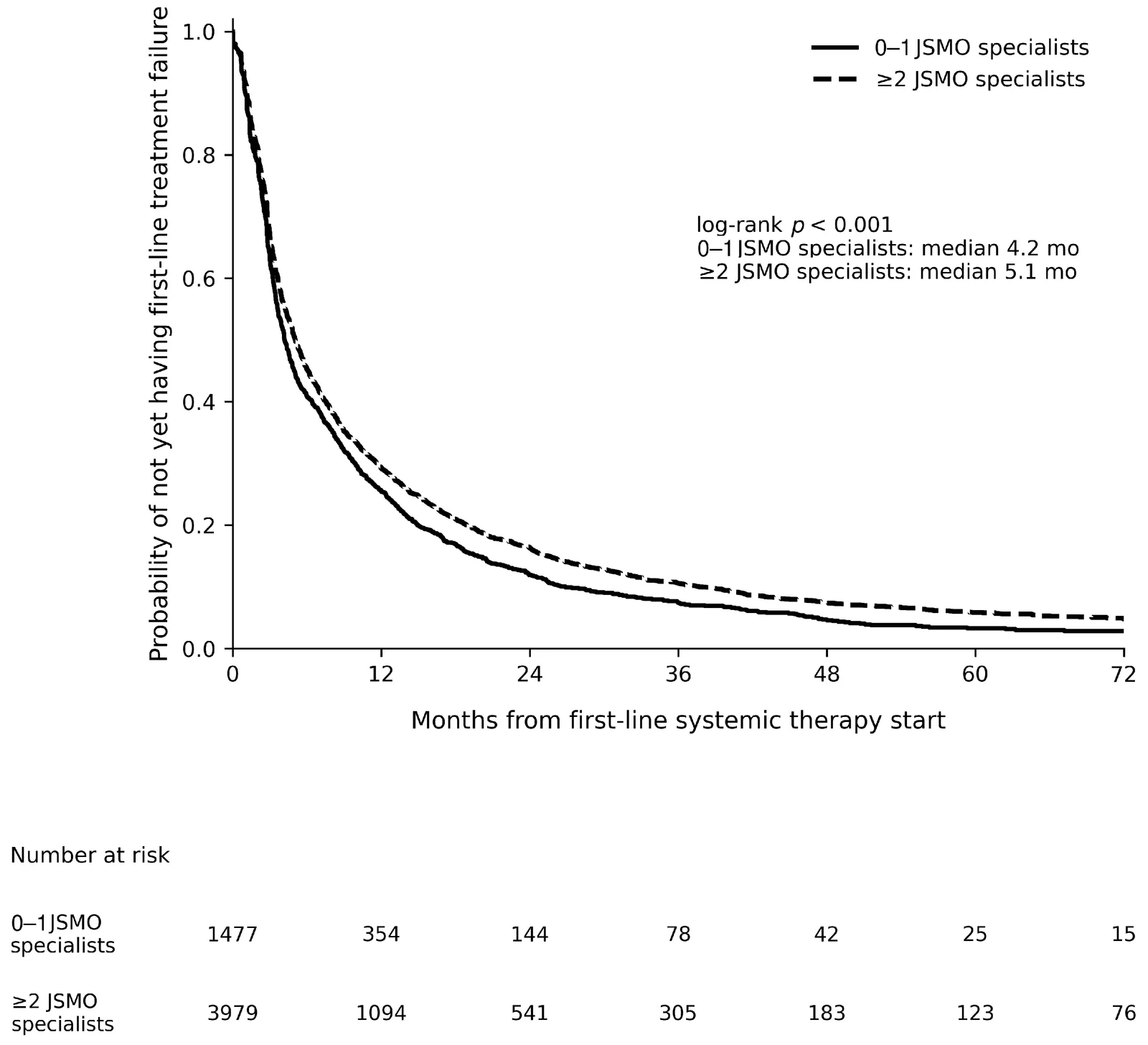
Kaplan–Meier curve for first-line time to treatment failure by facility-level JSMO specialist availability. Time zero was the first-line systemic therapy start date used for endpoint construction. First-line TTF was defined as time to recorded first-line treatment end or death, whichever occurred first; patients without either event were censored at the recorded non-death censoring date. Groups were defined as facilities with 0–1 versus ≥2 registry-listed JSMO specialists. Kaplan–Meier estimated median first-line TTF was 4.2 months for the 0–1 JSMO specialist group and 5.1 months for the ≥2 JSMO specialist group; log-rank *p* < 0.001. First-line TTF is a treatment-process endpoint and is not progression-free survival, treatment efficacy, survival benefit, facility quality, or evidence of patient-level specialist involvement. JSMO, Japanese Society of Medical Oncology; TTF, time to treatment failure.

Kaplan–Meier analysis showed longer first-line TTF at facilities with ≥2 registry-listed JSMO specialists than at facilities with 0–1 registry-listed JSMO specialists (Figure 1). Kaplan–Meier estimated median first-line TTF was 4.2 months in the 0–1 JSMO specialist group and 5.1 months in the ≥2 JSMO specialist group (log-rank *p* < 0.001).

### 3.4. Primary Cox Models

Using facility-cluster robust standard errors as the primary inferential basis, the HR for ≥2 versus 0–1 registry-listed JSMO specialists was 0.871 (robust 95% CI, 0.779–0.975; robust *p* = 0.016; *n* = 5456; events = 4967; 241 facility clusters) in Model 1, 0.944 (0.844–1.056; *p* = 0.315) in Model 2, and 0.937 (0.834–1.053; *p* = 0.275) in Model 3 (Table 2, Panel B). In complete-case clinical adjustment models, the HR was 0.907 (0.803–1.024; *p* = 0.115; *n* = 5188; events = 4748; 238 facility clusters) in Model 4 and 0.911 (0.806–1.029; *p* = 0.133; *n* = 5188; events = 4748; 238 facility clusters) in Model 5. Thus, the Model 5 point estimate was in the direction of longer observed first-line TTF, but the robust confidence interval included no association.

In the facility-volume sensitivity analysis, adding log2-transformed C-CAT strict-lung cohort volume to Model 5 yielded an exposure HR of 0.911 (robust 95% CI, 0.806–1.030; *p* = 0.138); the HR per doubling of facility volume was 0.995 (0.951–1.042; *p* = 0.843). There were 103 zero-time observations among all analyzable patients (32 in the 0–1 group and 71 in the ≥2 group); all represented recorded first-line treatment end on the start date. Model 5 results were unchanged after one-day replacement (HR, 0.911; 0.806–1.029; *p* = 0.133) and after excluding zero-time observations (HR, 0.912; 0.807–1.032; *p* = 0.143). Model 5 excluded 268 patients (4.9%): 69/1477 (4.7%) in the 0–1 group and 199/3979 (5.0%) in the ≥2 group. Multiple imputation was not performed because the prespecified histology unknown/missing group combined true blanks with clinically heterogeneous nonblank values. A sensitivity model retaining unknown/missing groups as explicit categories included all 5456 patients and yielded HR 0.922 (0.818–1.039; *p* = 0.183) (Supplementary Tables S5–S7).

Conventional ordinary Cox confidence intervals and *p* values are provided for comparison in Supplementary Table S4 and were not used as the primary inferential basis.

### 3.5. Secondary High-Confidence Dual JSMO/JRS Availability Analysis

High-confidence dual JSMO/JRS availability was evaluated as the prespecified secondary facility-level proxy for JSMO expertise oriented toward lung cancer care. In the primary analysis cohort, 2782 patients (51.0%) were treated at facilities without high-confidence dual JSMO/JRS availability, and 2674 patients (49.0%) were treated at facilities with high-confidence dual JSMO/JRS availability (Table 1). Baseline characteristics by high-confidence dual JSMO/JRS availability are shown in Supplementary Table S3.

**Figure 2:**
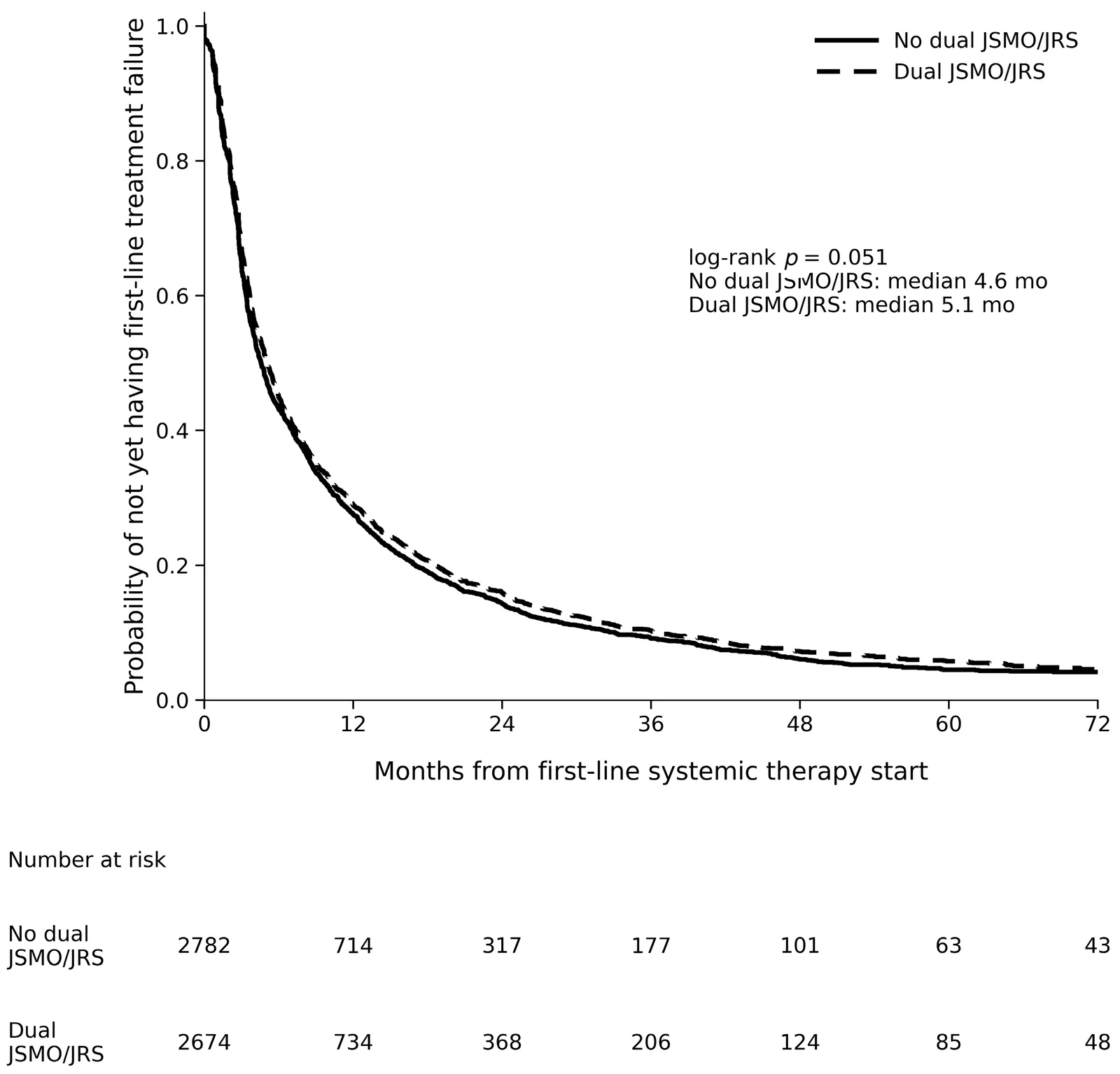
Kaplan–Meier curve for first-line time to treatment failure by high-confidence dual JSMO/JRS specialist availability. Time zero was the first-line systemic therapy start date used for endpoint construction. First-line TTF was defined as time to recorded first-line treatment end or death, whichever occurred first; patients without either event were censored at the recorded non-death censoring date. High-confidence dual JSMO/JRS availability denotes a facility-level registry-listed specialist matched as both JSMO and JRS. Kaplan–Meier estimated median first-line TTF was 4.6 months for facilities without high-confidence dual JSMO/JRS availability and 5.1 months for facilities with high-confidence dual JSMO/JRS availability; log-rank *p* = 0.051. This secondary figure describes unadjusted lung-cancer-facing facility-level structural context. First-line TTF is a treatment-process endpoint and should not be interpreted as progression-free survival, treatment efficacy, survival benefit, facility quality, dual-specialist management, or patient-level specialist involvement. JSMO, Japanese Society of Medical Oncology; JRS, Japanese Respiratory Society; TTF, time to treatment failure.

Kaplan–Meier analysis showed modest unadjusted separation by this secondary proxy (Figure 2). Kaplan–Meier estimated median first-line TTF was 4.6 months at facilities without high-confidence dual JSMO/JRS availability and 5.1 months at facilities with high-confidence dual JSMO/JRS availability (log-rank *p* = 0.051). Secondary Cox models attenuated after adjustment for genome medicine facility category and in complete-case clinical adjustment models; in the full clinical adjusted model, the HR was 0.995 (95% CI, 0.937–1.057; *p* = 0.868) (Supplementary Table S1).

### 3.6. Supportive Overall Survival Analyses

Supportive OS analyses are summarized in Supplementary Table S2 and Supplementary Figures S1 and S2. The endpoint-specific OS cohort contained 5349 analyzable observations; Model 5 included 5085 observations and 2393 deaths after 264 clinical complete-case exclusions. Ordinary Model 5 estimates were HR 1.133 (95% CI, 1.020–1.258; *p* = 0.020) for ≥2 versus 0–1 registry-listed JSMO specialists and HR 1.140 (95% CI, 1.047–1.241; *p* = 0.002) for dual versus no dual JSMO/JRS availability. Facility-cluster robust Model 5 inference in the estimable subset (*n* = 5084; 2393 deaths; 234 facility clusters) yielded HR 1.133 (robust 95% CI, 0.975–1.316; *p* = 0.102) and HR 1.140 (robust 95% CI, 1.013–1.283; *p* = 0.030), respectively. These OS analyses remained supportive and exploratory.

Among the 5456 patients in the current first-line TTF cohort, C-CAT registration occurred before, on the same day as, and after first-line start in 35, 4, and 5417 patients, respectively; the median interval was 621 days (IQR, 324–1172). The earliest available CGP specimen date occurred before, on the same day as, and after first-line start in 1879, 8, and 3563 patients, respectively, with 6 missing dates; the median interval was 350 days (IQR, −24 to 988). In the delayed-entry OS sensitivity, 20 observations with registration on or after exit and the single sparse-category observation were excluded. Among 5064 observations with 2391 deaths and 234 facility clusters, the HRs were 1.020 (robust 95% CI, 0.888–1.171; *p* = 0.780) for the primary JSMO exposure and 1.020 (robust 95% CI, 0.921–1.128; *p* = 0.707) for dual availability (Supplementary Table S10).

## 4. Discussion

### 4.1. Principal Findings

In this nationwide C-CAT lung cancer study, facilities with ≥2 registry-listed JSMO specialists had an adjusted point estimate in the direction of longer first-line TTF, but the primary facility-cluster robust confidence interval included no association. The secondary dual JSMO/JRS analysis provided a complementary lung-cancer-oriented view and showed no adjusted association with first-line TTF. By linking national CGP registry records with facility workforce data, this study demonstrates the feasibility of examining oncology-care infrastructure through an observable treatment-process endpoint. It also extends the health-services value of C-CAT beyond molecular characterization and identifies where more treatment-specific data are needed.

### 4.2. Interpretation as a Facility-Level Oncology Infrastructure Signal

Registry-listed JSMO specialist availability may function as a facility-level indicator of oncology-care infrastructure. In Japan, facilities with larger numbers of registry-listed JSMO specialists often have broader systemic anticancer therapy and cancer genomic medicine programs [6,7,10]. Formal genome medicine facility category captures part of this structure but may not fully reflect systemic therapy capacity, treatment-monitoring workflows, documentation practices, referral pathways, or the organization of genomic medicine [6,7,8]. Specialist availability may therefore capture aspects of the care context surrounding first-line systemic therapy rather than an isolated staffing variable. This interpretation is consistent with the expanding role of medical oncology in Japan and with C-CAT’s function as a national cancer genomic medicine infrastructure [7,10].

Specialist count and C-CAT strict-lung case volume may capture related but distinct features of facility infrastructure. They were moderately correlated, and adding log2-transformed volume did not materially change the exposure HR. Thus, this registry-derived volume variable did not explain the point-estimate direction, although staffing, referral, facility structure, and treatment selection remain intertwined.

### 4.3. Relevance to CGP-Era Real-World Oncology Practice

Linking a nationwide CGP registry with a facility-level workforce indicator extends C-CAT use from molecular and testing studies toward a health-services question. C-CAT has supported large-scale analyses of cancer genomic medicine, including the clinical utility of CGP in non-small cell lung cancer [3,7]. This linkage is particularly relevant in lung cancer, where molecular therapy, immunotherapy, cytotoxic therapy, specimen management, expert-panel workflow, and multidisciplinary coordination intersect [2,4,5]. Against this background, Fujii et al. analyzed 3240 cases registered from June 2019 through August 2023 and addressed molecular and testing utility in NSCLC, including companion-diagnostic-negative cases, tissue/plasma detection, and presumed germline findings [3]. The present study instead examines a facility-level health-services question using recorded first-line treatment-process duration. Some patients may overlap, but exact patient-level overlap cannot be determined from published aggregate data. The differing cohorts, extracts, questions, and endpoints make the studies complementary rather than an independent validation or replication.

### 4.4. Secondary Dual JSMO/JRS and Supportive OS Analyses

The high-confidence dual JSMO/JRS analysis served as a secondary, lung-cancer-oriented indicator rather than an alternative primary exposure. The presence of a registry-listed dual JSMO/JRS physician may capture respiratory-oncology-facing medical oncology expertise [4,5,10]. Dual availability showed modest unadjusted separation and no adjusted association with first-line TTF. It therefore provides complementary context on lung-cancer-facing facility infrastructure [14].

Ordinary supportive OS point estimates were in the direction opposite to the first-line TTF point estimate. With facility-cluster robust inference, the primary JSMO OS confidence interval included 1, whereas the dual-availability interval was above 1. Because the cohort was conditioned on reaching CGP/C-CAT, these patterns may reflect referral, case mix, CGP timing, and survival to registry inclusion [9,15]. When C-CAT registration was treated as delayed entry, both exposure estimates attenuated to approximately 1.02 and both robust confidence intervals included 1. Delayed entry refined risk sets after registration but could not restore patients who died before registry inclusion. Accordingly, these analyses provide complementary context rather than evidence of harm; first-line TTF and OS remain distinct outcomes [12,13].

### 4.5. Implications for Future National Clinical Databases

The findings translate current data gaps into concrete design requirements for future national clinical databases. A chemotherapy-specific database integrated with or complementary to C-CAT should capture patient-level specialist involvement, treatment-line transitions, reasons for discontinuation, toxicity and adverse events, dose modification, regimen details, treatment intent, time-anchored molecular testing, clinical trial access, and standardized follow-up [12,16,17]. These elements would connect care organization directly with treatment processes and outcomes rather than relying only on facility-level structural indicators [14].

### 4.6. Limitations

Several limitations concern the exposure and endpoint. Registry-listed specialist availability was a facility-level proxy and did not measure patient-level specialist involvement. The dual JSMO/JRS variable did not identify whether individual patients were managed by dual-certified physicians, reviewed in respiratory-oncology conferences, or treated within specific multidisciplinary workflows. Registry-listed affiliation may be incomplete, time-varying, or misclassified, and same-name matching may add error. First-line TTF was constructed from recorded treatment-line and death dates; it was not an imaging-based progression endpoint and should not be interpreted as progression-free survival, treatment efficacy, survival benefit, facility quality, or specialist contribution [12,13,16,17]. Its components have different clinical meanings, including planned completion, toxicity-related discontinuation, regimen change, and death. Appropriate earlier switching from an ineffective regimen could shorten TTF, whereas delayed switching could prolong it. The available first-line termination labels were coarse, did not separately identify radiographic progression, and were unknown, not recorded, or ambiguous for 8.2% of recorded-end events; detailed toxicity, dose modification, treatment intent, and standardized chemotherapy follow-up were unavailable [7,17].

First-line treatment class and driver-result fields were summarized descriptively but were not added to the Cox model because driver results lacked patient-level test dates and treatment class could lie downstream of the facility-level care process. Because TTF may differ by molecular subtype and treatment class, residual confounding by molecular profile, treatment selection, facility structure, referral, documentation, access, and patient selection remains possible [14]. JSMO specialists were concentrated in core or hub genomic medicine facilities, and formal facility category may not capture all relevant differences in systemic therapy infrastructure [6,7,10]. The primary cluster-robust adjusted interval included no association, underscoring cluster-aware uncertainty. The C-CAT strict-lung case count used for volume sensitivity was a study-specific registry volume, not institutional lung-cancer or chemotherapy volume. Model 5 excluded 268 patients because prespecified ECOG PS or histology groups were unknown/missing; overall exclusion proportions were similar by exposure, although variable-specific patterns differed. Multiple imputation was not performed because the histology unknown/missing group combined true blanks with clinically heterogeneous nonblank values; the retained-category sensitivity was consistent with the primary analysis.

The cohort was restricted to patients who underwent CGP and may not represent all patients with lung cancer receiving systemic therapy [3,7]. C-CAT registration occurred after first-line start for 99.3% of the current TTF cohort, so first-line-origin analyses condition on subsequent survival and referral to CGP and are susceptible to survivor-selection and delayed-entry mechanisms. The delayed-entry sensitivity addressed risk sets within the observed registered cohort but could not recover patients who died before CGP registration or remove referral and case-mix selection. The facility recorded for C-CAT/CGP was not verified as the site that initiated every first-line regimen. Patient residence, rurality, socioeconomic characteristics, and travel or referral distance were unavailable, preventing evaluation of related patient selection.

## 5. Conclusions

In this nationwide C-CAT cohort, the point estimate for facilities with ≥2 versus 0–1 registry-listed JSMO specialists was in the direction of longer recorded first-line treatment-process duration, while the facility-cluster robust confidence interval included no association. Registry-visible specialist availability can nonetheless provide a facility-level indicator for studying oncology-care infrastructure. Future national databases should link patient-level specialist involvement with time-anchored molecular data, regimen details, treatment transitions, and standardized follow-up so that care organization and treatment processes can be evaluated more directly.

## Figures and Tables

**Table 1:** Baseline characteristics of the primary analysis cohort by facility-level JSMO specialist availability.

Variable	Level/Statistic	Overall	0–1 JSMO Specialists	≥2 JSMO Specialists
Patients	N	5456	1477	3979
Facilities	N	241	97	144
Age	Median (IQR), years	66 (57–73)	66 (58–73)	66 (57–73)
Age group	<65 years	2443/5456 (44.8%)	645/1477 (43.7%)	1798/3979 (45.2%)
	65–74 years	2003/5456 (36.7%)	563/1477 (38.1%)	1440/3979 (36.2%)
	≥75 years	1010/5456 (18.5%)	269/1477 (18.2%)	741/3979 (18.6%)
Sex	Female	2029/5456 (37.2%)	534/1477 (36.2%)	1495/3979 (37.6%)
	Male	3427/5456 (62.8%)	943/1477 (63.8%)	2484/3979 (62.4%)
ECOG PS	0	2150/5456 (39.4%)	658/1477 (44.5%)	1492/3979 (37.5%)
	1	2893/5456 (53.0%)	711/1477 (48.1%)	2182/3979 (54.8%)
	≥2	334/5456 (6.1%)	76/1477 (5.1%)	258/3979 (6.5%)
	Unknown/missing	79/5456 (1.4%)	32/1477 (2.2%)	47/3979 (1.2%)
Lung histology/subtype	Adenocarcinoma	3459/5456 (63.4%)	970/1477 (65.7%)	2489/3979 (62.6%)
	Squamous cell carcinoma	607/5456 (11.1%)	154/1477 (10.4%)	453/3979 (11.4%)
	Small-cell lung cancer	377/5456 (6.9%)	96/1477 (6.5%)	281/3979 (7.1%)
	Large-cell/neuroendocrine carcinoma	154/5456 (2.8%)	41/1477 (2.8%)	113/3979 (2.8%)
	Other non-small-cell lung cancer	655/5456 (12.0%)	179/1477 (12.1%)	476/3979 (12.0%)
	Unknown/missing	204/5456 (3.7%)	37/1477 (2.5%)	167/3979 (4.2%)
Specimen type	Tissue	3912/5456 (71.7%)	990/1477 (67.0%)	2922/3979 (73.4%)
	Blood/liquid	1542/5456 (28.3%)	486/1477 (32.9%)	1056/3979 (26.5%)
	Other	2/5456 (0.0%)	1/1477 (0.1%)	1/3979 (0.0%)
Panel type	FoundationOne CDx	3334/5456 (61.1%)	899/1477 (60.9%)	2435/3979 (61.2%)
	FoundationOne Liquid CDx	1361/5456 (24.9%)	415/1477 (28.1%)	946/3979 (23.8%)
	NCC Oncopanel	420/5456 (7.7%)	78/1477 (5.3%)	342/3979 (8.6%)
	Guardant360	193/5456 (3.5%)	73/1477 (4.9%)	120/3979 (3.0%)
	GenMineTOP	148/5456 (2.7%)	12/1477 (0.8%)	136/3979 (3.4%)
Treatment start year	Median (IQR)	2021 (2019–2022)	2021 (2019–2022)	2021 (2019–2022)
Genome medicine facility category	Core hospital	856/5456 (15.7%)	0/1477 (0.0%)	856/3979 (21.5%)
	Hub hospital	1460/5456 (26.8%)	99/1477 (6.7%)	1361/3979 (34.2%)
	Liaison hospital	3140/5456 (57.6%)	1378/1477 (93.3%)	1762/3979 (44.3%)
JRS specialist availability	0–1 JRS specialists	88/5456 (1.6%)	19/1477 (1.3%)	69/3979 (1.7%)
	≥2 JRS specialists	5368/5456 (98.4%)	1458/1477 (98.7%)	3910/3979 (98.3%)
High-confidence dual JSMO/JRS availability	No dual JSMO/JRS	2782/5456 (51.0%)	1311/1477 (88.8%)	1471/3979 (37.0%)
	Dual JSMO/JRS	2674/5456 (49.0%)	166/1477 (11.2%)	2508/3979 (63.0%)
Facility has both JSMO and JRS availability	No combined JSMO/JRS availability	579/5456 (10.6%)	573/1477 (38.8%)	6/3979 (0.2%)
	Combined JSMO/JRS availability	4877/5456 (89.4%)	904/1477 (61.2%)	3973/3979 (99.8%)
Smoking status	Never smoker	1590/5456 (29.1%)	438/1477 (29.7%)	1152/3979 (29.0%)
	Ever smoker	3773/5456 (69.2%)	1019/1477 (69.0%)	2754/3979 (69.2%)
	Unknown/missing	93/5456 (1.7%)	20/1477 (1.4%)	73/3979 (1.8%)

Values are *n*/N (%) unless otherwise specified. The primary analysis cohort includes cases analyzable for the first-line TTF endpoint. Unknown/missing rows are shown for variables with incomplete data. Percentages use the primary analysis cohort denominator within each column unless otherwise specified. ECOG PS, lung histology/subtype, and smoking status may have missing or unknown values. JSMO denotes the Japanese Society of Medical Oncology; JRS denotes the Japanese Respiratory Society; ECOG PS denotes Eastern Cooperative Oncology Group performance status; TTF denotes time to treatment failure. JSMO and JRS specialist availability variables are facility-level, registry-listed availability proxies and do not indicate direct patient-level specialist involvement. High-confidence dual JSMO/JRS availability denotes the presence of a registry-listed specialist matched as both JSMO and JRS at the facility. Genome medicine facility category was grouped as core, hub, or liaison hospitals. Specimen type was grouped as tissue, blood/liquid, or other. Panel type was normalized to FoundationOne CDx, FoundationOne Liquid CDx, NCC Oncopanel, Guardant360, or GenMineTOP. No *p* values are shown.

**Table 2:** Primary endpoint ascertainment and Cox models for first-line time to treatment failure.

**Panel A. First-Line TTF Ascertainment by Facility-Level JSMO Specialist Availability**						
**Metric**	**0–1 JSMO Specialists**	**≥2 JSMO Specialists**		**Interpretive Note**		
C-CAT-defined lung cancer cases	1580	4158		Pre-endpoint-exclusion denominator.		
Facilities	98	145		C-CAT-defined denominator; analyzable facilities: 97 and 144.		
First-line TTF analyzable	1477/1580 (93.5%)	3979/4158 (95.7%)		Used as the primary endpoint denominator.		
First-line TTF events among analyzable	1370/1477 (92.8%)	3597/3979 (90.4%)		Recorded first-line treatment end or death, whichever occurred first.		
Censored among analyzable	107/1477 (7.2%)	382/3979 (9.6%)		No recorded first-line treatment end or death; censored at the recorded non-death censoring date.		
Negative interval	3/1580 (0.2%)	8/4158 (0.2%)		Excluded from the primary endpoint denominator.		
Excluded from primary denominator	103/1580 (6.5%)	179/4158 (4.3%)		Missing dates or negative intervals.		
Observed time to event or censoring, median (IQR), months	4.2 (2.3–11.5)	5.0 (2.5–13.4)		Median (IQR) among primary endpoint analyzable cases.		
**Panel B. Cox models with primary facility-cluster robust inference**						
**Exposure**	**Model**	**HR**	**Robust 95% CI**	**Robust *p* value**	***n*** **/events**	**Facility clusters**
≥2 vs. 0–1 registry-listed JSMO specialists	Model 1: unadjusted	0.871	0.779–0.975	0.016	5456/4967	241
	Model 2: facility-category adjusted	0.944	0.844–1.056	0.315	5456/4967	241
	Model 3: facility-category + treatment-start-year adjusted	0.937	0.834–1.053	0.275	5456/4967	241
	Model 4: core clinical adjusted	0.907	0.803–1.024	0.115	5188/4748	238
	Model 5: full clinical adjusted	0.911	0.806–1.029	0.133	5188/4748	238

First-line TTF was defined as time from first-line systemic therapy start to recorded first-line treatment end or death, whichever occurred first. Patients without a recorded first-line treatment end or death were censored at the recorded non-death censoring date. Events included recorded first-line treatment end or death before a recorded treatment end; patients without either event were censored. First-line TTF is a treatment-process endpoint and is not progression-free survival, treatment efficacy, survival benefit, facility quality, or evidence of patient-level specialist involvement. In Panel B, HR < 1 indicates a lower hazard of first-line treatment failure and corresponds to longer observed time without recorded first-line treatment failure. Primary 95% CIs and *p* values were estimated using facility-cluster robust standard errors based on treating facility code. Model 1 was unadjusted; Model 2 adjusted for genome medicine facility category; Model 3 adjusted for genome medicine facility category and treatment start year; Model 4 adjusted for age, sex, ECOG PS, histology, genome medicine facility category, and treatment start year; and Model 5 adjusted for age, sex, ECOG PS, histology, specimen type, panel type, genome medicine facility category, and treatment start year. Models 4 and 5 are complete-case clinical adjustment models. Conventional ordinary Cox inference is shown in Supplementary Table S4. Panel A reports empirical event-or-censoring medians; Kaplan–Meier product-limit medians account for censoring.

## Data Availability

The authors do not control the underlying C-CAT database. The anonymized C-CAT data analyzed in this study were obtained through the approved C-CAT data-use framework and are subject to institutional and data-provider governance restrictions. Accordingly, the source-level data are not publicly available from the authors. Access to C-CAT data requires a formal application to C-CAT and approval by the relevant institutional and data-use review bodies under the Japanese cancer genomic medicine governance framework.

## Supplementary Materials

The following supporting information can be downloaded at: https://www.mdpi.com/article/10.3390/curroncol33080462/s1, Table S1: Secondary Cox models for selected alternative facility-level specialist availability exposures for first-line TTF; Table S2: Supportive overall survival Cox models for selected facility-level specialist availability exposures; Table S3: Baseline characteristics by high-confidence dual JSMO/JRS availability; Table S4: Conventional ordinary Cox models for the primary JSMO specialist-count exposure; Table S5: Facility-volume description and volume-adjusted sensitivity analysis; Table S6: Zero-time audit and sensitivity analyses; Table S7: Complete-case missingness and retained-category sensitivity analysis; Figure S1: Supportive overall survival by facility-level JSMO specialist availability; Figure S2: Supportive overall survival by high-confidence dual JSMO/JRS availability. Table S8: Endpoint-event source, censoring, and termination-reason availability; Table S9: First-line treatment class and driver-status distribution; Table S10: CGP-timing and time-origin sensitivity analyses for supportive overall survival.
