# Supplementary material for: Facility-Level Medical Oncology Specialist Availability and First-Line Time to Treatment Failure in Lung Cancer: A Nationwide C-CAT Registry Analysis
Source: Curr Oncol. 2026 Aug 1;33(8):462. doi: 10.3390/curroncol33080462 (PMC13511204; doi:10.3390/curroncol33080462)
Supplement: Supplementary file 1 [file curroncol-33-00462-s001.zip › 38lung_CO_Supplementary_20260719d.pdf]

Supplementary Materials

Supplementary Table S1. Secondary Cox models for selected alternative facility-level specialist availability exposures for first-line TTF

Exposure	Comparison	Model	HR	95% CI	*p* value	N/events
High-confidence dual JSMO/JRS availability	Dual JSMO/JRS vs no dual JSMO/JRS	Model 1	0.946	0.895–1.000	0.052	5456/4967
		Model 2	1.001	0.944–1.061	0.983	5456/4967
		Model 3	1.018	0.960–1.079	0.553	5456/4967
		Model 4	0.993	0.935–1.054	0.815	5188/4748
		Model 5	0.995	0.937–1.057	0.868	5188/4748
Exploratory ≥4 versus 0–3 JSMO specialist availability threshold	≥4 vs 0–3 registry-listed JSMO specialists	Model 1	0.892	0.843–0.944	<0.001	5456/4967
		Model 2	1.029	0.960–1.104	0.418	5456/4967
		Model 3	1.070	0.998–1.148	0.057	5456/4967
		Model 4	1.056	0.982–1.134	0.141	5188/4748
		Model 5	1.040	0.968–1.118	0.286	5188/4748
Combined JSMO/JRS facility availability	Combined JSMO/JRS availability vs no combined JSMO/JRS availability	Model 1	0.905	0.827–0.990	0.029	5456/4967
		Model 2	0.978	0.890–1.075	0.646	5456/4967
		Model 3	0.981	0.893–1.078	0.690	5456/4967
		Model 4	0.936	0.850–1.030	0.173	5188/4748
		Model 5	0.934	0.849–1.028	0.162	5188/4748

First-line TTF was defined as the time from first-line systemic therapy start to recorded first-line treatment end or death, whichever occurred first. HR < 1 indicates a lower hazard of recorded first-line treatment failure. Specialist variables are facility-level registry-listed availability proxies and do not indicate patient-level specialist involvement or facility quality. First-line TTF should not be interpreted as progression-free survival, treatment efficacy, or survival benefit. Model 1 was unadjusted; Model 2 adjusted for genome medicine facility category; Model 3 adjusted for genome medicine facility category and treatment start year; Model 4 adjusted for age, sex, ECOG PS, histology, genome medicine facility category, and treatment start year; and Model 5 adjusted for age, sex, ECOG PS, histology, specimen type, panel type, genome medicine facility category, and treatment start year. Models 4 and 5 are complete-case clinical adjustment models.

TTF, time to treatment failure.

Supplementary Table S2. Supportive overall survival Cox models for selected facility-level specialist availability exposures

Exposure	Comparison	Model	HR	Ordinary 95% CI; p	Facility-cluster robust 95% CI; p	N/deaths (ordinary)	Facilities
**Facility-level JSMO specialist availability**	≥2 vs 0–1 registry-listed JSMO specialists	Model 1	1.264	1.151–1.389; <0.001	—	5349/2502	237
		Model 2	1.245	1.123–1.382; <0.001	—	5349/2502	237
		Model 3	1.224	1.104–1.357; <0.001	—	5349/2502	237
		Model 4	1.132	1.020–1.258; 0.020	—	5085/2393	234
		Model 5	1.133	1.020–1.258; 0.020	0.975–1.316; 0.102	5085/2393	234
**High-confidence dual JSMO/JRS availability**	Dual JSMO/JRS vs no dual JSMO/JRS	Model 1	1.157	1.070–1.252; <0.001	—	5349/2502	237
		Model 2	1.132	1.042–1.230; 0.003	—	5349/2502	237
		Model 3	1.228	1.131–1.333; <0.001	—	5349/2502	237
		Model 4	1.145	1.052–1.246; 0.002	—	5085/2393	234
		Model 5	1.140	1.047–1.241; 0.002	1.013–1.283; 0.030	5085/2393	234
**Combined JSMO/JRS facility availability**	Combined JSMO/JRS availability vs no combined JSMO/JRS availability	Model 1	1.347	1.165–1.557; <0.001	—	5349/2502	237
		Model 2	1.297	1.115–1.508; <0.001	—	5349/2502	237
		Model 3	1.240	1.066–1.442; 0.005	—	5349/2502	237
		Model 4	1.193	1.024–1.390; 0.024	—	5085/2393	234
		Model 5	1.197	1.027–1.396; 0.021	0.915–1.568; 0.190	5085/2393	234
**Exploratory ≥4 versus 0–3 JSMO specialist availability threshold**	≥4 vs 0–3 registry-listed JSMO specialists	Model 1	1.206	1.115–1.304; <0.001	—	5349/2502	237
		Model 2	1.227	1.111–1.356; <0.001	—	5349/2502	237
		Model 3	1.415	1.285–1.559; <0.001	—	5349/2502	237
		Model 4	1.326	1.200–1.466; <0.001	—	5085/2393	234
		Model 5	1.278	1.155–1.413; <0.001	1.138–1.435; <0.001	5085/2393	234

OS was a supportive/exploratory endpoint and was not the primary endpoint. HRs are unchanged ordinary Cox coefficients. Ordinary confidence intervals and p values are shown for comparison; Model 5 facility-cluster robust confidence intervals and p values use treating-facility clusters and the estimable 5084-case subset (2393 deaths; 234 facilities). The ordinary Model 5 cohort contained 5085 observations; one censored specimen-type "other" observation produced a nonestimable nuisance coefficient and was excluded only for robust inference, without changing the exposure coefficients at the reported precision. Facility-cluster robust estimates were calculated for Model 5 only; therefore, Models 1–4 show ordinary estimates only. Model 5 adjusted for age, sex, ECOG PS, histology, specimen type, panel type, genome medicine facility category, and treatment start year. Time zero used the recorded first-line start when available and otherwise the earliest recorded systemic-therapy administration start. The event was the earliest parseable death date; non-death observations were censored at the latest parseable last-survival-confirmation date. OS results should not be used to infer treatment efficacy, causal benefit or harm, facility quality, or patient-level specialist involvement.

OS, overall survival; TTF, time to treatment failure.

Supplementary Table S3. Baseline characteristics by high-confidence dual JSMO/JRS availability

Variable	Level/statistic	Overall	No dual JSMO/JRS	Dual JSMO/JRS
Patients	N	5456	2782	2674
Facilities	N	241	151	90
Age	Median (IQR), years	66 (57–73)	66 (57–73)	66 (56–73)
Age group	<65 years	2443/5456 (44.8%)	1259/2782 (45.3%)	1184/2674 (44.3%)
	65–74 years	2003/5456 (36.7%)	1004/2782 (36.1%)	999/2674 (37.4%)
	≥75 years	1010/5456 (18.5%)	519/2782 (18.7%)	491/2674 (18.4%)
Sex	Female	2029/5456 (37.2%)	1042/2782 (37.5%)	987/2674 (36.9%)
	Male	3427/5456 (62.8%)	1740/2782 (62.5%)	1687/2674 (63.1%)
ECOG PS	0	2150/5456 (39.4%)	1241/2782 (44.6%)	909/2674 (34.0%)
	1	2893/5456 (53.0%)	1314/2782 (47.2%)	1579/2674 (59.1%)
	≥2	334/5456 (6.1%)	174/2782 (6.3%)	160/2674 (6.0%)
	Unknown/missing	79/5456 (1.4%)	53/2782 (1.9%)	26/2674 (1.0%)
Lung histology/subtype	Adenocarcinoma	3459/5456 (63.4%)	1795/2782 (64.5%)	1664/2674 (62.2%)
	Squamous cell carcinoma	607/5456 (11.1%)	296/2782 (10.6%)	311/2674 (11.6%)
	Small-cell lung cancer	377/5456 (6.9%)	193/2782 (6.9%)	184/2674 (6.9%)
	Large-cell/neuroendocrine carcinoma	154/5456 (2.8%)	87/2782 (3.1%)	67/2674 (2.5%)
	Other non-small-cell lung cancer	655/5456 (12.0%)	343/2782 (12.3%)	312/2674 (11.7%)
	Unknown/missing	204/5456 (3.7%)	68/2782 (2.4%)	136/2674 (5.1%)
Specimen type	Tissue	3912/5456 (71.7%)	1944/2782 (69.9%)	1968/2674 (73.6%)
	Blood/liquid	1542/5456 (28.3%)	836/2782 (30.1%)	706/2674 (26.4%)
	Other	2/5456 (0.0%)	2/2782 (0.1%)	0/2674 (0.0%)
Panel type	FoundationOne CDx	3334/5456 (61.1%)	1717/2782 (61.7%)	1617/2674 (60.5%)
	FoundationOne Liquid CDx	1361/5456 (24.9%)	748/2782 (26.9%)	613/2674 (22.9%)
	NCC Oncopanel	420/5456 (7.7%)	141/2782 (5.1%)	279/2674 (10.4%)
	Guardant360	193/5456 (3.5%)	98/2782 (3.5%)	95/2674 (3.6%)
	GenMineTOP	148/5456 (2.7%)	78/2782 (2.8%)	70/2674 (2.6%)
Treatment start year	Median (IQR)	2021 (2019–2022)	2021 (2019–2022)	2020 (2019–2022)
Genome medicine facility category	Core hospital	856/5456 (15.7%)	248/2782 (8.9%)	608/2674 (22.7%)
	Hub hospital	1460/5456 (26.8%)	501/2782 (18.0%)	959/2674 (35.9%)
	Liaison hospital	3140/5456 (57.6%)	2033/2782 (73.1%)	1107/2674 (41.4%)
Facility has both JSMO and JRS availability	No combined JSMO/JRS availability	579/5456 (10.6%)	579/2782 (20.8%)	0/2674 (0.0%)
	Combined JSMO/JRS availability	4877/5456 (89.4%)	2203/2782 (79.2%)	2674/2674 (100.0%)

Values are *n*/N (%) unless otherwise specified. Unknown/missing rows are shown where applicable. Percentages use the denominator shown in each column. No *p* values are shown. Dual JSMO/JRS availability and combined JSMO/JRS availability are facility-level registry-listed availability proxies and do not indicate patient-level specialist involvement, dual-specialist management, multidisciplinary care delivery, treatment efficacy, progression-free survival, survival benefit, or facility quality.

JSMO, Japanese Society of Medical Oncology; JRS, Japanese Respiratory Society; ECOG PS, Eastern Cooperative Oncology Group performance status.

Supplementary Table S4. Conventional ordinary Cox models for the primary JSMO specialist-count exposure

Model	HR	Ordinary 95% CI	Ordinary *p* value	*n*/events	Facilities
Model 1: unadjusted	0.871	0.819–0.927	<0.001	5456/4967	241
Model 2: facility-category adjusted	0.944	0.882–1.011	0.098	5456/4967	241
Model 3: facility-category + treatment-start-year adjusted	0.937	0.876–1.003	0.063	5456/4967	241
Model 4: core clinical adjusted	0.907	0.846–0.972	0.006	5188/4748	238
Model 5: full clinical adjusted	0.911	0.849–0.976	0.008	5188/4748	238

Conventional ordinary Cox confidence intervals and *p* values are shown for comparison. Primary inference for the primary facility-level exposure used facility-cluster robust standard errors in Table 2.

Supplementary Table S5. Facility-volume description and volume-adjusted sensitivity analysis

Exposure group	Facilities	Patients	Facility-level median (IQR)	Range	Patient-weighted median (IQR)
0–1 JSMO specialists	98	1580	9 (4–23.75)	1–74	35 (17–50)
≥2 JSMO specialists	145	4158	20 (8–37)	1–269	48 (27–71)
Overall	243	5738	15 (5–33)	1–269	42 (25–64)

Sensitivity term	HR	Robust 95% CI	Robust *p* value	*n*/events/clusters
≥2 vs 0–1 JSMO specialists	0.911	0.806–1.030	0.138	5188/4748/238
Facility volume per doubling	0.995	0.951–1.042	0.843	5188/4748/238

Facility volume was the C-CAT strict-lung cohort case count per facility before primary-endpoint analyzability filtering; it is not institutional lung-cancer volume or chemotherapy volume. Facility-level JSMO specialist count and facility volume were moderately correlated (Spearman $\rho = 0.342$; $p < 0.001$), with overlapping volume ranges. The sensitivity model added log2-transformed volume to Model 5 and used facility-cluster robust standard errors.

Supplementary Table S6. Zero-time audit and sensitivity analyses

Audit metric	Overall	0–1 JSMO specialists	≥2 JSMO specialists
Zero-time observations, all analyzable	103	32	71
Zero-time observations, Model 5	97	30	67
Event reason among zero-time observations	Recorded first-line end (103)	Recorded first-line end (32)	Recorded first-line end (71)
Smallest positive observed interval	1 day	—	—

Zero-time rule	*n*/events/clusters	HR	Robust 95% CI	Robust *p* value
Primary 1e-6-month offset	5188/4748/238	0.911	0.806–1.029	0.133
Replace zero with 1 day	5188/4748/238	0.911	0.806–1.029	0.133
Exclude zero-time observations	5091/4651/238	0.912	0.807–1.032	0.143

Source dates had day-level values. All zero-time observations represented a recorded first-line treatment end on the same date as treatment start. Every prespecified alternative is reported; the prespecified primary model remained the primary analysis.

Supplementary Table S7. Complete-case missingness and retained-category sensitivity analysis

**Variable**	**Overall**	**0–1 JSMO specialists**	**≥2 JSMO specialists**	**Classification**
ECOG PS unknown/missing	79/5456 (1.4%)	32/1477 (2.2%)	47/3979 (1.2%)	58 explicit Unknown; 21 raw blanks
Histology other/unknown	204/5456 (3.7%)	37/1477 (2.5%)	167/3979 (4.2%)	43 both raw fields blank; 161 nonblank structural other/unknown
Any Model 5 complete-case exclusion	268/5456 (4.9%)	69/1477 (4.7%)	199/3979 (5.0%)	ECOG PS and/or histology
**Sensitivity approach**	***n*/events/clusters**	**HR**	**Robust 95% CI**	**Robust *p* value**
Unknown/missing groups retained as explicit categories	5456/4967/241	0.922	0.818–1.039	0.183

Multiple imputation was considered but not performed. The prespecified histology other/unknown group combined true blanks with rare histologies, broad or ambiguous labels, typographic variants, and unmapped nonblank values. Imputing all 204 as one homogeneous missing-data state would require redesigning the frozen covariate mapping and could invent unavailable clinical information. The alternative retained these groups explicitly without imputing clinical categories.

Supplementary Table S8. Endpoint-event source, censoring, and termination-reason availability

**Endpoint category**	**Overall (N = 5456)**	**0–1 JSMO specialists (N = 1477)**	**≥2 JSMO specialists (N = 3979)**
Recorded first-line treatment end	4680 (85.8%)	1336 (90.5%)	3344 (84.0%)
Death without a recorded first-line treatment end	287 (5.3%)	34 (2.3%)	253 (6.4%)
Censored observation	489 (9.0%)	107 (7.2%)	382 (9.6%)
Total	5456 (100%)	1477 (100%)	3979 (100%)

Percentages use the full primary-endpoint cohort within each column. Current endpoint-event totals were 4680 recorded first-line end events, 287 deaths without a recorded first-line end, and 489 censored observations.

**Explicit termination-reason source label**	**Overall (N = 4680)**	**0–1 JSMO specialists (N = 1336)**	**≥2 JSMO specialists (N = 3344)**
Invalidation cancellation	2370 (50.6%)	643 (48.1%)	1727 (51.6%)
Finished as planned	1151 (24.6%)	417 (31.2%)	734 (21.9%)
Canceled due to side effects, etc.	605 (12.9%)	165 (12.4%)	440 (13.2%)
Canceled for other reasons	122 (2.6%)	35 (2.6%)	87 (2.6%)
Canceled at the request of the person	46 (1.0%)	12 (0.9%)	34 (1.0%)
Not recorded	289 (6.2%)	35 (2.6%)	254 (7.6%)
Unknown	96 (2.1%)	28 (2.1%)	68 (2.0%)
Ambiguous: multiple recorded reasons	1 (<0.1%)	1 (0.1%)	0 (0.0%)
Total recorded-end events	4680 (100%)	1336 (100%)	3344 (100%)

Termination-reason percentages use recorded first-line treatment-end events within each column. A reason was used only when an explicit first-line record ended on the endpoint date; no reason was inferred. “Invalidation cancellation” is the exact source label and was not reclassified as radiographic progression. Death without a recorded first-line end is an endpoint source, not an inferred discontinuation reason.

Supplementary Table S9. First-line treatment class and driver-status distribution

Mutually exclusive first-line treatment class	Overall (N = 5456)	0–1 JSMO specialists (N = 1477)	≥2 JSMO specialists (N = 3979)
Cytotoxic chemotherapy plus immune checkpoint inhibitor	2004 (36.7%)	562 (38.1%)	1442 (36.2%)
Cytotoxic chemotherapy without immune checkpoint inhibitor	1980 (36.3%)	577 (39.1%)	1403 (35.3%)
Immune checkpoint inhibitor therapy without cytotoxic chemotherapy	340 (6.2%)	95 (6.4%)	245 (6.2%)
Molecularly targeted therapy/TKI	745 (13.7%)	200 (13.5%)	545 (13.7%)
Other systemic therapy	100 (1.8%)	10 (0.7%)	90 (2.3%)
Other/mixed systemic therapy	36 (0.7%)	10 (0.7%)	26 (0.7%)
Unknown/unclassifiable	251 (4.6%)	23 (1.6%)	228 (5.7%)
Total	5456 (100%)	1477 (100%)	3979 (100%)

All records explicitly labeled first-line treatment were aggregated, including maintenance or additions within that labeled line. Classification hierarchy was targeted plus cytotoxic/immune checkpoint inhibitor (mixed), targeted alone, immune checkpoint inhibitor plus cytotoxic, immune checkpoint inhibitor without cytotoxic, cytotoxic without immune checkpoint inhibitor, other systemic therapy, then unknown/unclassifiable. Across non-mutually-exclusive mechanism flags, cytotoxic therapy was recorded in 4018/5456 (73.6%), targeted therapy in 781/5456 (14.3%), and immune checkpoint inhibitor-containing therapy in 2351/5456 (43.1%).

Available driver-result summary	Overall (N = 5456)	0–1 JSMO specialists (N = 1477)	≥2 JSMO specialists (N = 3979)
At least one documented positive result	1066 (19.5%)	302 (20.4%)	764 (19.2%)
No documented positive; ≥1 explicit negative result	3538 (64.8%)	976 (66.1%)	2562 (64.4%)
No positive or negative result documented	852 (15.6%)	199 (13.5%)	653 (16.4%)
C-CAT registration after first-line start	5417 (99.3%)	1470 (99.5%)	3947 (99.2%)
All available CGP specimens after first-line start	3563 (65.3%)	993 (67.2%)	2570 (64.6%)
≥1 CGP specimen on/before first-line start	1887 (34.6%)	483 (32.7%)	1404 (35.3%)
CGP specimen date unavailable	6 (0.1%)	1 (0.1%)	5 (0.1%)

Eight fields were available: EGFR, ALK, ROS1, BRAF V600, MET exon 14, KRAS G12C, RET, and NTRK1/2/3 fusion. Positive counts were 804, 124, 70, 17, 34, 22, 12, and 2, respectively; patients could have more than one positive field. “No documented positive; ≥1 explicit negative” is not equivalent to a complete negative driver panel. Unknown/not tested, negative, missing, and indeterminate values were retained as separate categories in the analysis dataset. Biomarker-result test dates were unavailable; CGP specimen dates did not establish when separate lung biomarker results were obtained. Because biomarker-result test dates were unavailable and CGP specimen dates did not establish the timing of separate lung biomarker testing, a baseline driver-status covariate could not be defined reliably; therefore, no treatment-class/driver-adjusted Cox model was fitted.

Supplementary Table S10. CGP-timing and time-origin sensitivity analyses for supportive overall survival

Panel A summarizes calendar-day timing relative to first-line start. Panel B compares ordinary first-line-origin and C-CAT-registration delayed-entry Model 5 analyses.

Component	Group/exposure	Definition/comparison	*n*/deaths/clusters	HR	Robust 95% CI	Robust p
CGP timing	Overall	C-CAT registration before / same day / after first-line start: 35 / 4 / 5417; median 621 days (IQR, 324–1172)	5456/—/241	—	—	—
	0–1 JSMO specialists	C-CAT registration before / same day / after: 7 / 0 / 1470; median 665 days (IQR, 359–1214)	1477/—/97	—	—	—
	≥2 JSMO specialists	C-CAT registration before / same day / after: 28 / 4 / 3947; median 609 days (IQR, 308–1158)	3979/—/144	—	—	—
CGP timing	Overall	Earliest specimen before / same day / after first-line start: 1879 / 8 / 3563; 6 missing; median 350 days (IQR, −24 to 988)	5456/—/241	—	—	—
	0–1 JSMO specialists	Earliest specimen before / same day / after: 483 / 0 / 993; 1 missing; median 403 days (IQR, −22 to 1054)	1477/—/97	—	—	—
	≥2 JSMO specialists	Earliest specimen before / same day / after: 1396 / 8 / 2570; 5 missing; median 331 days (IQR, −25 to 961)	3979/—/144	—	—	—
Time-origin model	≥2 vs 0–1 JSMO specialists	First-line origin, robust estimable subset	5084/2393/234	1.133	0.975–1.316	0.102
	≥2 vs 0–1 JSMO specialists	Matched 5064-case first-line-origin model	5064/2391/234	1.134	0.977–1.316	0.099
	≥2 vs 0–1 JSMO specialists	C-CAT registration delayed entry; (entry, exit]	5064/2391/234	1.020	0.888–1.171	0.780
Time-origin model	Dual vs no dual JSMO/JRS	First-line origin, robust estimable subset	5084/2393/234	1.140	1.013–1.283	0.030
	Dual vs no dual JSMO/JRS	Matched 5064-case first-line-origin model	5064/2391/234	1.136	1.010–1.278	0.033
	Dual vs no dual JSMO/JRS	C-CAT registration delayed entry; (entry, exit]	5064/2391/234	1.020	0.921–1.128	0.707

Delayed entry retained first-line start as the time-scale origin and used C-CAT registration as cohort entry. Sixteen same-day entry/exit observations and four observations with registration after exit were excluded because they could not contribute a positive risk interval; the single censored sparse specimen-type “other” observation was also excluded for estimable robust Model 5 inference. Registration and outcome dates were available at day precision without time-of-day. Delayed entry addresses observed risk-set construction after C-CAT entry but cannot recover patients who died before CGP/C-CAT inclusion or remove referral and case-mix selection. JSMO and JRS exposures remain facility-level availability proxies.

Supplementary Figure legends

Supplementary Figure S1. Supportive overall survival by facility-level JSMO specialist availability. Time zero used the same first-line systemic therapy start-date rule as the primary endpoint: the recorded first-line start when available, otherwise the earliest recorded systemic therapy administration start. The event was death, and cases without a death event were censored at the recorded last survival-confirmation or censoring date. Groups were defined as facilities with 0–1 versus ≥2 registry-listed JSMO specialists. Median OS was 60.0 months for facilities with 0–1 JSMO specialists and 49.1 months for facilities with ≥2 JSMO specialists; log-rank $p < 0.001$. This supportive OS analysis was not the primary endpoint and should not be used to infer treatment efficacy, survival benefit or harm, facility quality, causal specialist effect, or patient-level specialist involvement.

JSMO, Japanese Society of Medical Oncology; OS, overall survival.

Supplementary Figure S2. Supportive overall survival by high-confidence dual JSMO/JRS availability. Time zero used the same first-line systemic therapy start-date rule as the primary endpoint: the recorded first-line start when available, otherwise the earliest recorded systemic therapy administration start. The event was death, and cases without a death event were censored at the recorded last survival-confirmation or censoring date. High-confidence dual JSMO/JRS availability denotes a facility-level registry-listed specialist matched as both JSMO and JRS. Median OS was 54.7 months for facilities without high-confidence dual JSMO/JRS availability and 49.1 months for facilities with high-confidence dual JSMO/JRS availability; log-rank $p < 0.001$. This supportive OS analysis was not the primary endpoint and should not be used to infer treatment efficacy, survival benefit or harm, facility quality, causal specialist effect, patient-level specialist involvement, or dual-specialist management.

JSMO, Japanese Society of Medical Oncology; JRS, Japanese Respiratory Society; OS, overall survival.
