# Supplementary material for: Facility-Level Medical Oncology Specialist Availability and First-Line Time to Treatment Failure in Lung Cancer: A Nationwide C-CAT Registry Analysis
Source: Curr Oncol. 2026 Aug 1;33(8):462. doi: 10.3390/curroncol33080462 (PMC13511204; doi:10.3390/curroncol33080462)
Supplement: Supplementary file 1 [file curroncol-33-00462-s001.zip › 38lung_FigureS1_20260510a.pdf]

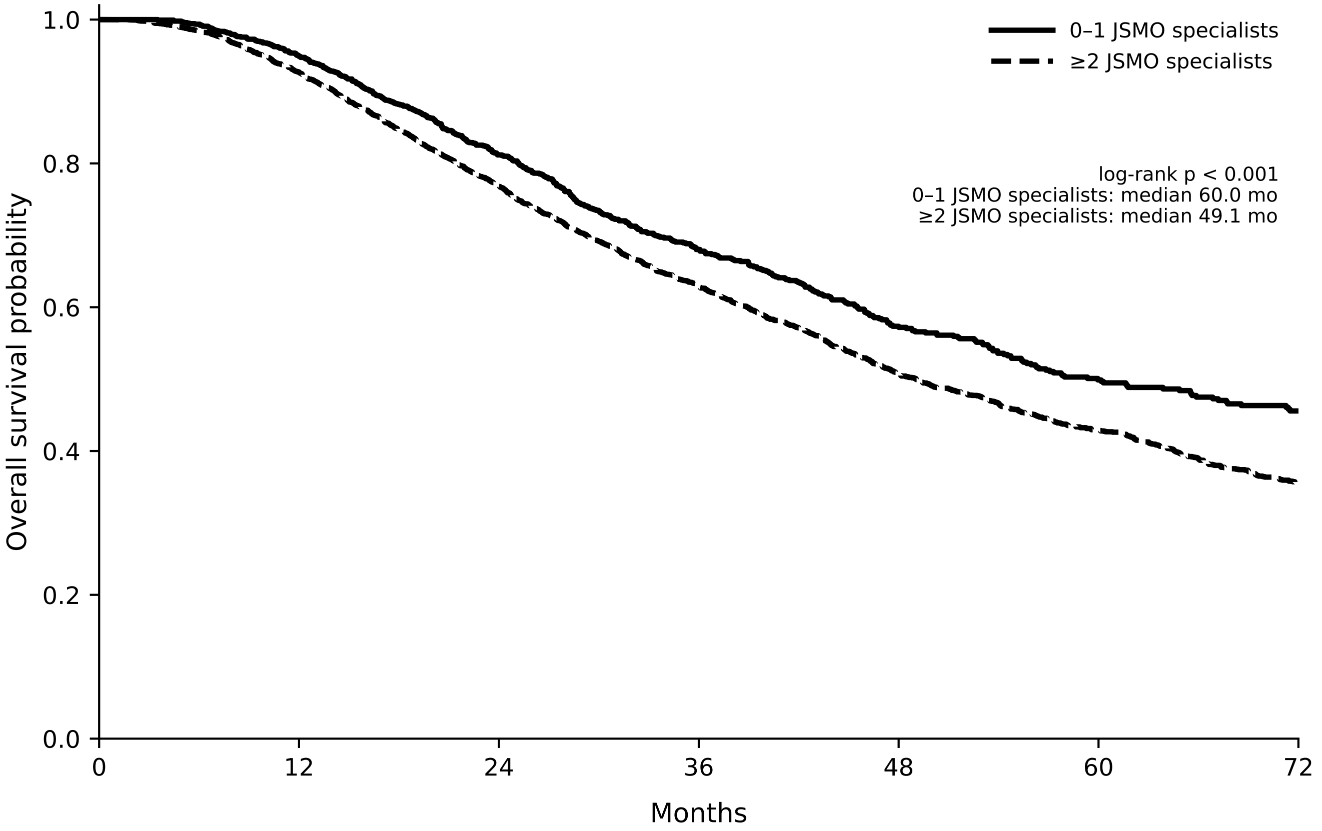

Number at risk

	0	12	24	36	48	60	72
0–1 JSMO specialists	1420	1261	888	587	379	246	181
≥2 JSMO specialists	3929	3395	2384	1622	1070	751	486
